# Loss-of-function variant in spermidine/spermine N1-acetyl transferase like 1 (*SATL1*) gene as an underlying cause of autism spectrum disorder

**DOI:** 10.1038/s41598-024-56253-5

**Published:** 2024-03-08

**Authors:** Abdulfatah M. Alayoubi, Muhammad Iqbal, Hassan Aman, Jamil A. Hashmi, Laila Alayadhi, Khalid Al-Regaiey, Sulman Basit

**Affiliations:** 1 Department of Basic Medical Sciences, Taibah University Medina, Almadinah Almunawwarah, Saudi Arabia; 2 Department of Physiology, Faculty of Medicine, King Saud University, Riyadh, Saudi Arabia; 3 Al-Amal Psychiatry Hospital Medina, Almadinah Almunawwarrah, Saudi Arabia; 4 Center for Genetics and Inherited Diseases, Taibah University Medina, Almadinah Almunawwarrah, Saudi Arabia; 5 Autism Research and Treatment Center, Riyadh, Saudi Arabia; 6 Department of Basic Medical Sciences, Taibah University Medina, Almadinah Almunawwarrah, Saudi Arabia

**Keywords:** Late onset ASD, Polyamines, Mutation, Exome sequencing, Clinical genetics, Genomics, Medical genetics, Mutation, Neurodevelopmental disorders, Genetics

## Abstract

Autism spectrum disorder (ASD) is a complicated, lifelong neurodevelopmental disorder affecting verbal and non-verbal communication and social interactions. ASD signs and symptoms appear early in development before the age of 3 years. It is unlikely for a person to acquire autism after a period of normal development. However, we encountered an 8-year-old child who developed ASD later in life although his developmental milestones were normal at the beginning of life. Sequencing the complete coding part of the genome identified a hemizygous nonsense mutation (NM_001367857.2):c.1803C>G; (p.Tyr601Ter) in the gene (*SATL1*) encoding spermidine/spermine N1-acetyl transferase like 1. Screening an ASD cohort of 28 isolated patients for the *SATL1* gene identified another patient with the same variant. Although *SATL1* mutations have not been associated with any human diseases, our data suggests that a mutation in *SATL1* is the underlying cause of ASD in our cases. In mammals, mutations in spermine synthase (*SMS*), an enzyme needed for the synthesis of spermidine polyamine, have been reported in a syndromic form of the X-linked mental retardation. Moreover, *SATL1* gene expression studies showed a relatively higher expression of *SATL1* transcripts in ASD related parts of the brain including the cerebellum, amygdala and frontal cortex. Additionally, spermidine has been characterized in the context of learning and memory and supplementations with spermidine increase neuroprotective effects and decrease age-induced memory impairment. Furthermore, spermidine biosynthesis is required for spontaneous axonal regeneration and prevents α-synuclein neurotoxicity in invertebrate models. Thus, we report, for the first time, that a mutation in the *SATL1* gene could be a contributing factor in the development of autistic symptoms in our patients.

## Introduction

Autism spectrum disorder (ASD) is a heterogeneous neurodevelopmental disorder characterized by impairment in social communication and restricted and stereotyped behavior in early childhood^1^. Recent estimates suggest that more than 2% of people are affected with ASD worldwide^2,3^. The onset of ASD occurs during early age and is more prevalent in males than females (3:1 male-to-female ratio) in the general population^4^. Although autism is diagnosed early in childhood, an increasing body of evidence suggests that many individuals are diagnosed for the first time later on in life^5^. There are many possible reasons for the late diagnosis such as having less severe symptoms^6^, a higher IQ, being female by gender^7^, having less educated parents^8^, or the overlapping of autism symptoms with other psychiatric conditions^9^. There is also a possibility that at the time of diagnosis in childhood, symptoms of other conditions such as attention-deficit hyperactivity disorder^10^ or obsessive–compulsive disorder^11^ may have overshadowed the autism symptoms.

There is no single cause of autism, but genetic, environmental and immunological factors contribute to the etiology of ASD. The genetic aspect of autism was noted earlier by Kanner, however, twin and family studies explained the heritability aspect of ASD^12,13^. The biological family members of autistic children, not meeting the criteria for an autism diagnosis, also showed communication and social interaction dificulties^14^. A survey of more than 2 million Swedish children estimated the heritability contribution to be 50%, stressing on the important role of genetic factors in the etiology of ASD^15^.

Advancement in gene technologies such as whole genome sequencing, whole-exome sequencing and microarrays have provided a better understanding of genetic contribution to ASD. Genetic changes such as copy number variations, single nucleotide polymorphism and de novo mutations play a role in the pathophysiology of ASD^16^. The genes known to be involved in ASD pathobiology converge on common biological mechanisms such as transcription and signaling pathways^17^, synapse formation, synapse transmission and synapse plasticity^18^, epigenetic regulation^19^, and biological pathways related to neuro-inflammatory response^20^.

A large exome sequencing study of 35,584 individuals, including 11,986 with ASD, identified 102 disease risk genes^21^. Functionally the identified genes were involved in the regulation of gene expression, neuronal communication and cytoskeletal organization during early brain development in ASD. Analysis of the data revealed gain-of-function mutations in the genes named *DEAF1*, *SC1A*, *SLC6A1* and *KCNQ3* in the potassium channel. The authors also proposed a new driver gene *BCL11A* in copy number variations in ASD^21^.

Polyamines are organic compounds with two or more than two amino groups present in prokaryotic and eukaryotic cells. They are involved in many cellular functions, such as cellular proliferation, apoptosis and DNA, RNA and protein synthesis^22^. In mammals, spermidine, a ubiquitously present polyamine, is synthesized by spermidine synthase. Genetic mutations in this enzyme have been reported in a syndromic form of the X-linked mental retardation disorder, Snyder-Robinson syndrome^23–26^. In this study we report, for the first time, a rare genetic mutation in the spermidine/spermine N1-Acetyltransferase Like 1 (*SATL1)* gene in ASD patients.

## Materials and methods

### Recruitment of patients with ASD phenotype

**Figure 1:**
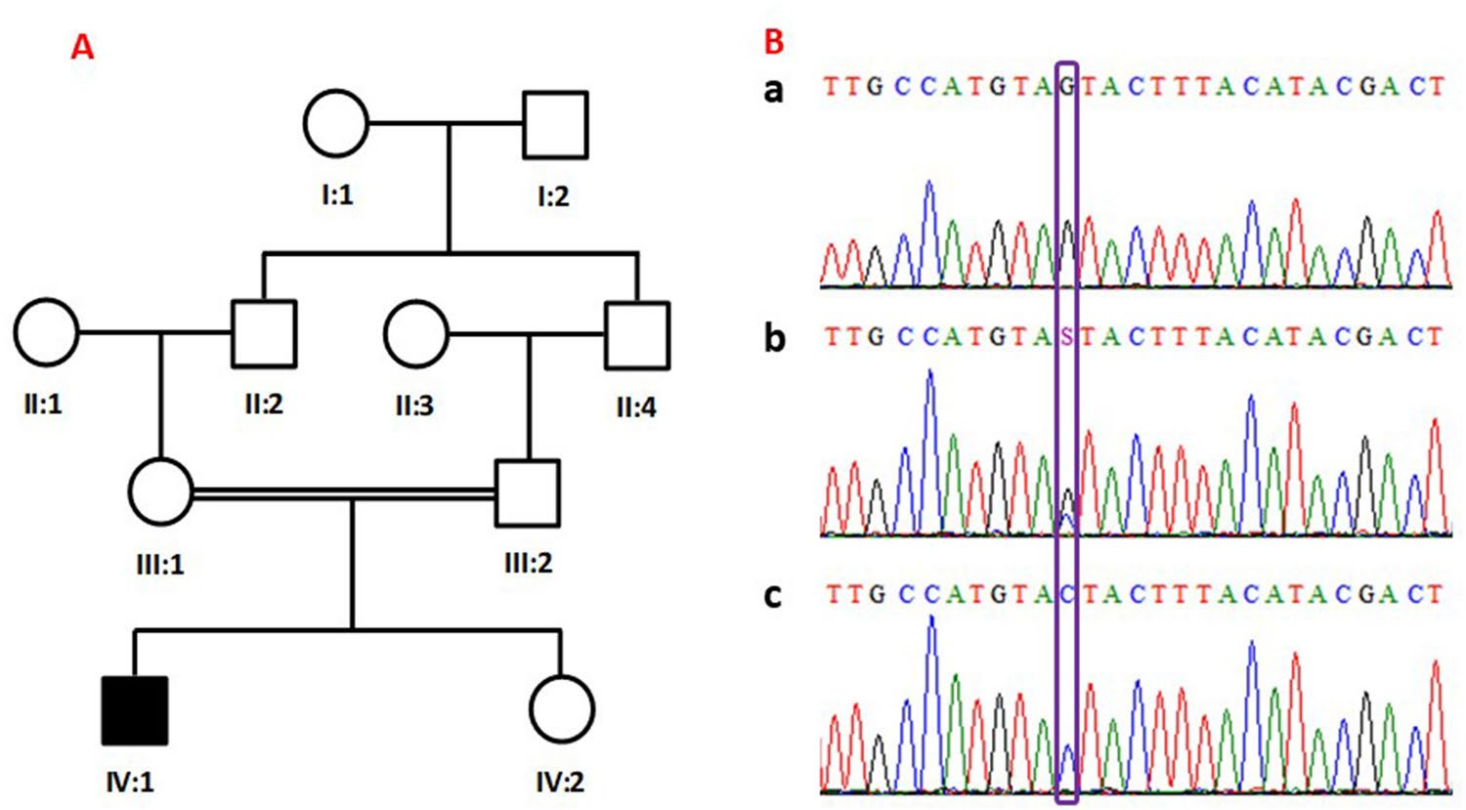
A pedigree with a proband. Square represent male individuals and circles represent female individuals. Filled sign show affected individual (**A**). Partial sequence chromatogram of a *SATL1* gene (**B**) in affected individuals (a), parents (b), and unaffected individuals and controls (c).

A family having a child with ASD features visited the Al-Amal Psychiatry Hospital in the Madinah region of the Kingdom of Saudi Arabia. A psychiatrist clinically evaluated the affected male proband (IV:1). The parents were interviewed and a pedigree chart was drawn (Fig. 1A). Twenty-eight isolated ASD patients from a separate cohort were also included in this study. These cases included broad spectrum autism phenotype (BAP) related cases as well as confirmed ASD cases. Written informed consents for genetic analysis were obtained from/for all the participants. The Research Ethics Committee of Taibah University (TUCD-REC/20151013/SAMMAN) approved the study. All experimental work was carried out according to the declaration of Helsinki. Blood samples from the affected individual (IV:1), unaffected individual (IV:2) and both parents (III:1 and III:2) were collected in EDTA containing vacutainers.

### DNA extraction and exome sequencing

Genomic DNA was extracted using a QIAquick DNA extraction kit (Qiagen, Manchester UK). DNA was visualized on 2% agarose gel and quantified using a spectrophotometer. DNA samples of both affected individuals were subjected to whole exome sequencing (WES) to comprehensively analyze the exonic regions of the genomes. WES was performed using Illumina DNA Prep with an Exome 2.0 Plus Enrichment kit. Paired-end sequencing (2 × 150 bp) was performed on the NextSeq2000 instrument (Illumina San Diego CA). Raw reads were aligned to the human reference genome (GRCh38) using BWA-MEM algorithm^27^. Duplicates were marked using Picard version 1.125^28^. Single nucleotide variants (SNVs) were called using SAMtools version 0.1.19 and short indels were called using Platypus version 0.7.4^29,30^. Homozygosity mapping using AutoMap was used to detect regions of homozygosity shared by affected individuals only^31^. Regions with homozygosity scores > 90% of the maximum and > 0.5 Mb in size were investigated further. All regions with > 0.5 Mb of homozygosity shared by affected individuals were considered candidate regions for the phenotype. Variants were functionally classified using wANNOVAR with gene model definitions from Gencode version v19^32^. Variants with frequencies < 0.01 in all public databases including gnomAD, ESP6500 and 1000 Genomes were evaluated. Homozygous, compound heterozygous, hemizygous, exonic or splicing variants, and those variants not present in our 130 in-lab exomes were selected. Primers were designed for variants of interest. Sanger sequencing was carried out on all available family members as well as the isolated ASD cases to validate the variants of interest.

## Results

### Clinical findings in affected individuals

Affected individual (IV:1), aged 10, was clinically assessed by the pediatric psychiatrist et al.-Amal hospital. The child (IV:1) presented to the outpatient department of the child psychiatry at the age of 8 years. He was referred by the primary care physician with complaints of clear regression in speech, social interaction, and academic performance and a decline in daily living activities at home within the last 3 months. The sudden onset of these symptoms at the age of eight years was quite puzzling, therefore, we initiated the psychiatric assessment.

#### Medical history

A caesarian section was performed for the delivery of the child (IV:1) because he was considered “large for gestational age” at gestational week 39 (approximately 3.5 kg). Hypoxia had not been reported at the time of birth and he did not require any ICU stay. Audiometry at the time of birth was within the normal range. Normal gross and fine motor skills were observed. He achieved all developmental milestones and no delay was reported in his speech. Normal speech development accompanied by appropriate nonverbal communication was attained at the age of 2 years. He was toilet trained at an appropriate age. At 1 year of age, he had febrile convulsions and was diagnosed with tonic–clonic epilepsy. His epileptic fits were preceded by an aura and clear post-ictal somnolence and a few hours of sleep. He was started on sodium valproate by the neurologist. His early education was in a regular school and no issues were reported at school. His academic performance was above average.

#### Psychiatric assessment

At the age of 8 years during assessment at the child psychiatry clinic he did not respond to his name and avoided eye contact. He preferred playing alone to playing with the assessor, did not share well or take turns and was not interested in interacting or socializing with others. Additionally, he did not know how to make friends. A major delay in speech was also noticed. He performed repetitive motions (flaps hands, rocks back and forth, spins on the floor at home). His mother reported that he would get upset or frustrated by small changes in his daily routine and had obsessive interests. He had major difficulty following daily routines at home. There were no self-harming behaviors reported at home, however, when irritable, he would often break household items. He was not known to have any food or medication allergies. There was no family history of any psychiatry issues. On physical examination, microcephaly, facial abnormalities, prominent ears, nystagmus and lymphedema were not observed. His neurological examination was within the normal range including all reflexes.

The child underwent a full psychological assessment which included an IQ assessment, an autism spectrum disorder assessment and an attention deficit disorder assessment. His IQ was 42 based on the Vineland Adaptive Behaviors Scales (Vineland-3). The ASD assessment and final diagnosis were based on the clinical opinion of an expert multidisciplinary team. He scored high on the (CARS™2) Childhood Autism Rating Scale™, Second Edition. He also received an additional diagnosis of attention deficit hyperactivity disorder. He scored high on The Connors rating scale which correlated with the clinical observations of the treatment psychiatrist. He was started on Risperidone and Atomoxetine drugs alongside the sodium valproate and L-carnitine for his epilepsy. He is responding to current medications and the disruptive behaviors at home have decreased. His sleep and appetite are within the normal range. He attends follow up appointments at the child psychiatry unit every three months and is monitored for metabolic side effects from the ongoing medication. He also attends a training center for Autism and receives speech and occupational therapy regularly.

The late onset of ASD features in this child prompted us to perform an exome wide scan to identify the underlying pathogenic variant.

### Genetic analysis revealed a protein truncating variant in the *SATL1* gene

**Figure 2:**
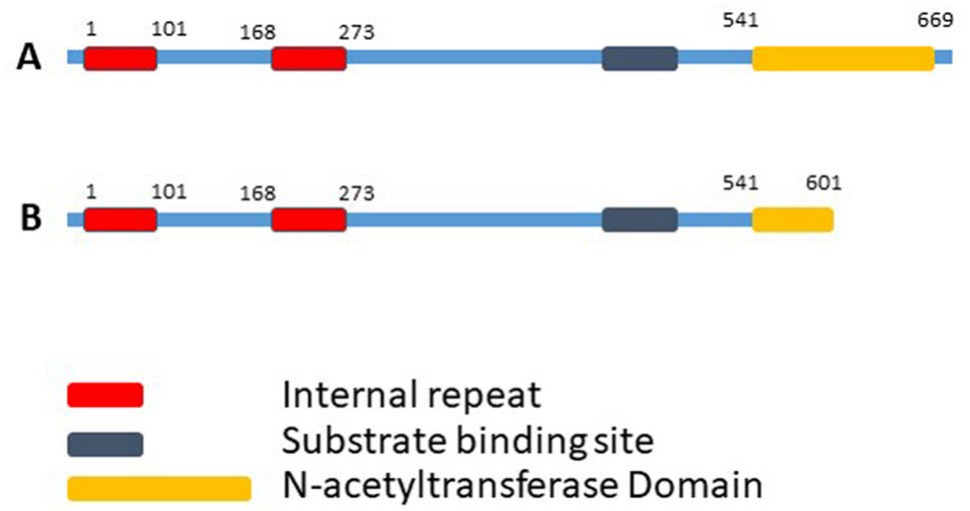
Schematic representation of SATL1 protein domains. Complete protein with all domains (**A**). Protein with partial acetyltransferase domain due to nonsense mutation in the acetyltransferase domain (**B**).

**Figure 3:**
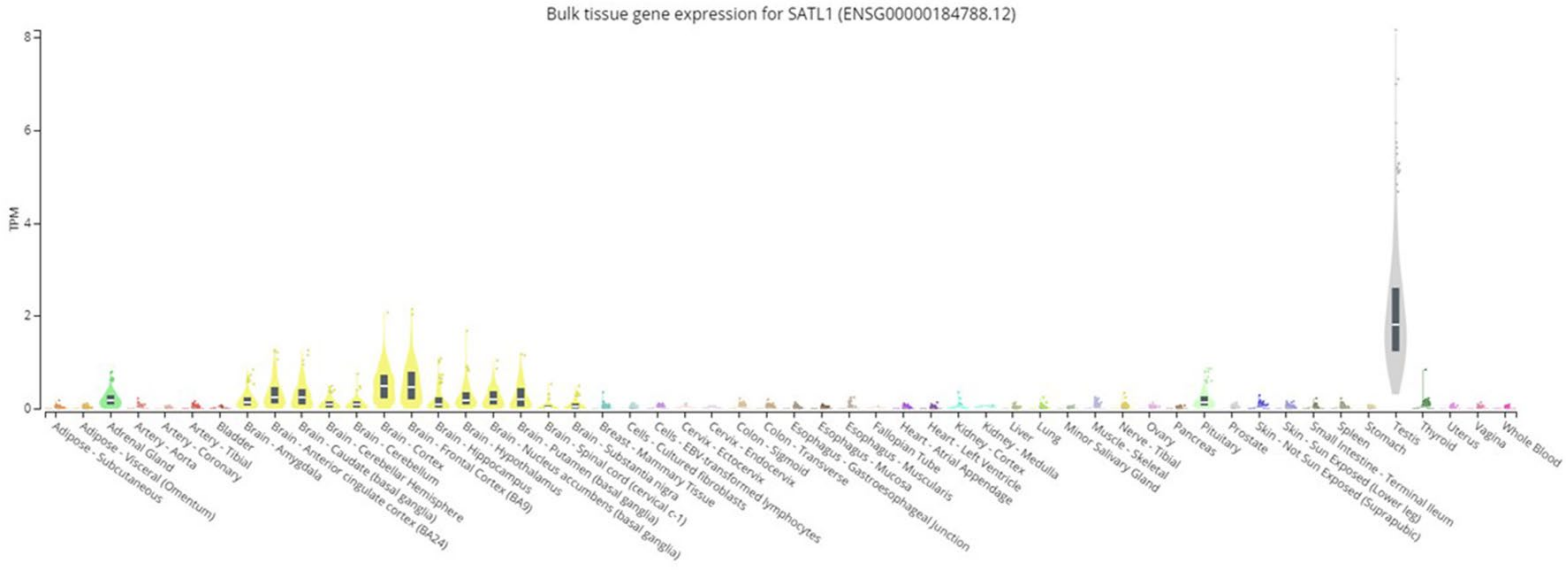
Tissue expression analyses showing high expression of *SATL1* transcripts in the testes and in different parts of the brain including the cerebellum, amygdala, cortex and frontal cortex. Data has been taken from https://gtexportal.org/home/gene/SATL1.

**Figure 4:**
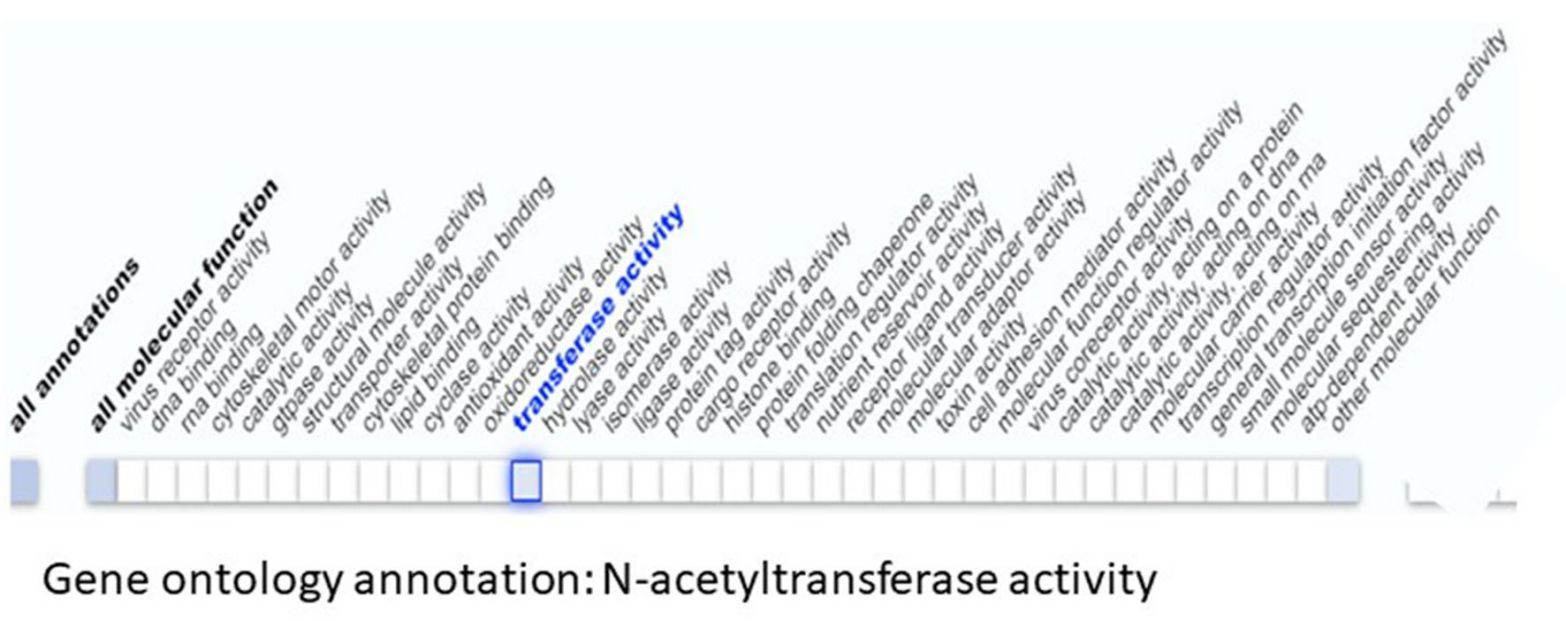
Gene ontology analysis showing the *SATL1* protein expression in the cytosol and its major function as *N*-acetyltransferase. GO analysis also shows that SATL1 protein binds to spermidine. Data has been taken from https://www.uniprot.org/uniprotkb/Q86VE3/entry.

Cutting-edge sequencing technology with advanced bioinformatics analysis was used to elucidate genetic variant(s) across the exomes of both samples. Analyses of the exome sequencing data and variant filtration led to the prioritization of 11 rare variants. Variants related to the disease features were prioritized and further investigated. A novel C to G substitution (c.1803C>G) in exon 6 of the *SATL1* gene was considered a strong candidate variant. Sanger sequencing validated the presence of the variant in the hemizygous state in both patients (Fig. 1B). Sequencing the flanking region in all available individuals showed that the variant was maternally segregating (Fig. 1B). Genomic DNA from twenty-eight ASD patients was screened for the complete coding part of the *SATL1* gene. This revealed a similar variant (c.1803C>G) in another patient with late onset ASD. The variant was not found in the 130 in-house exomes and was absent in the online human genome diversity panels gnomAD, HGMD and 1000 Genome databases. SATL1 protein has substrate binding and acetyltransferase domains (Fig. 2A). The nonsense variant (NM_001367857.2; c.1803C>G) is predicted stop gain (p.Tyr601Ter) and the protein product is predicted to lose partial acetyltransferase domain (Fig. 2B). Tissue expression analyses (https://gtexportal.org/home/gene/SATL1) have shown a high expression of *SATL1* in the testes and in different parts of the brain including the cerebellum, amygdala, cortex and frontal cortex. (Fig. 3). Moreover, Gene ontology (GO) analysis (https://www.uniprot.org/uniprotkb/Q86VE3/entry) showed that the *SATL1* protein expresses in the cytosol and performs N-acetyltransferase activity (Fig. 4).

## Discussion

Late-onset ASD features in a grown-up child are rare. It has been shown that many autistic people go undiagnosed throughout childhood and only learn that they have autism in adulthood^33^. This may be due to a misdiagnosis or due to the late recognition of autism. Children with less recognizable traits can go undiagnosed and some autistic individuals learn masking strategies to cope with their autistic traits. However, in our study, both affected individuals had normal developmental milestones as per their parents and physicians. Moreover, they were studying and performing well at school. At the age of around 8 years, they started developing ASD traits. They were diagnosed using Vineland-3. The Vineland Adaptive Behaviors Scales specifically measures adaptive behavior skills in communication, daily living skills, socialization and motor skills. While commonly used on children and adolescents, The Vineland-3 considers perspectives from an individual’s parent, caregiver and/or teacher. et al.-Amal Hospital, the ASD assessment utilizes multiple measures and methodologies (e.g., structured and semi-structured measures, parent report and direct observation) and the final diagnosis is based on the clinical opinion of an expert multidisciplinary team. A sudden deterioration in all aspects of language, behavior and academic achievement was observed at the age of 9 years, and so an initial diagnosis of ASD, ADHD or a learning disability was suspected.

In this study, we identified a hemizygous loss-of-function variant (c.1803C>G) in the spermidine/spermine N1-acetyl transferase like 1 (*SATL1*) encoding gene in a patient from a Saudi family. Segregation analysis showed that the variant in this gene is maternally segregating (Fig. 1B). Screening a cohort of isolated ASD cases identified another male ASD patient with the same variant. Interestingly, the clinical history of this patient revealed that the ASD features started appearing at the age of 7 years in his case and that he was given an ASD diagnosis at the age of 10. His birth was unremarkable with normal developmental milestones. Gross as well as fine motor skills were normal. He has normal speech development and appropriate nonverbal communication. The variant, identified in both the cases, introduces a premature termination codon (PTC) in the acyl transferase domain of the *SATL1* protein. The PTC is predicted to activate a non-sense mediated decay (NMD) pathway. This is supported by the models that posit that the degradation of PTC-containing mRNA by NMD requires the presence of PTCs located > 50–55nt or > 54–60nt upstream of the 3ʹ-most exon-exon junction^34,35^. Although tissue expression studies have shown a high expression of transcripts in the testes, the *SATL1* gene (GTEx) showed relatively higher expression in ASD related parts of the brain including the cerebellum, amygdala and frontal cortex^36^. These brain regions have been involved in driving ASD-related symptoms^37^. Moreover, the *SATL1* protein expresses in the cytosol and performs *N*-acetyltransferase activity as well as binding to spermidine^38^.

The contribution of the cerebellum in ASD includes impairments in social interaction, posture and gait control^39^. In ASD, cerebellum dysfunction disrupts the remote neocortical circuits and leads to a certain phenotype of ASD^40^. The amygdala is one of the earliest structures reported for its role in ASD. The abnormal growth pattern of the amygdala starts during 6–12 months of age, prior to the onset of the behavioral symptoms that are diagnostic for ASD^41^. The abnormal growth pattern of the amygdala and the dysregulation of amygdala function account for clinical features of ASD like impairment in social interaction, intangible cognition, planning and decision making and facial and emotional recognition^37^. The frontal cortex regulates many executive functions of the brain such as high-order cognitive functions including decision making, complex cognitive behaviors, leaning and communication^36^. Neuroimaging studies have shown that alterations in the cortical architecture lead to ASD symptoms such as impairment in social and communication behavior^42^, and motor^43^ and sensory difficulties^44^.

Spermidine biosynthesis is required for spontaneous axonal regeneration and prevents α-synuclein neurotoxicity in invertebrate models^45,46^. Moreover, spermidine reduced frontotemporal lobar dementia (FTLD-U) and improved the recognition memory deficit in a rodent model of Huntington’s disease^47,48^. Furthermore, it improves the spatial learning and memory capabilities of old mice^49^. Dietary spermidine supplementation ameliorated age-induced memory impairment in flies and protected from autoimmune-directed demyelination of neurons in a mouse model for multiple sclerosis^50^.

Polyamines including spermidine and spermine are ubiquitously occurring and are essential for cell growth, proliferation and tissue regeneration. They bind to and stabilize DNA and RNA, have antioxidative activities, modulate enzyme functions, and are required for the regulation of translation^22,51,52^. Polyamines have also been characterized in the context of learning and memory^53^. Increased neuroprotective effects, decreased age-induced memory impairment and positive effects on synaptic active zones are induced by dietary or otherwise externally applied spermidine supplements^50^.

Although the role of *SATL1* in spermidine biosynthesis is unknown, we hypothesize that the neuroprotective actions of spermidine are due to its interaction with *SATL1* followed by an increased autophagy in neuronal and glial cells. Patients deficient in *SATL1* might experience neurodegeneration. This is supported by the notion that polyamine and arginine metabolism is disturbed in mouse models of Alzheimer’s disease^54^ and in human patients with mild cognitive impairments (MCIs)^55^. It is further supported by the observation that several metabolites including spermidine were differentially affected in stable MCI patients and patients that later developed Alzheimer’s disease^55^. Thus, we report, for the first time, mutations in the *SATL1*/spermidine gene in ASD patients, which could be one of the contributing factors in the development of ASD symptoms in our patients. Future studies are needed to detect the potential causality between alterations in spermidine metabolism and neurodevelopmental diseases and their progression. Moreover, additional studies are required to see whether spermidine dietary supplementation reverses the pathological neurodegenerative disorders in humans.

## Data Availability

The datasets generated during and/or analysed during the current study are available in the European Variation Archive (EVA) repository. The accession number is PRJEB48950 and the link to data is (https://www.ebi.ac.uk/eva/?Study-Browser&browserType=sgv).
